# Clinical malaria incidence and health seeking pattern in geographically heterogeneous landscape of western Kenya

**DOI:** 10.1186/s12879-022-07757-w

**Published:** 2022-10-03

**Authors:** Wilfred Ouma Otambo, Patrick O. Onyango, Kevin Ochwedo, Julius Olumeh, Shirley A. Onyango, Pauline Orondo, Harrysone Atieli, Ming-Chieh Lee, Chloe Wang, Daibin Zhong, Andrew Githeko, Guofa Zhou, John Githure, Collins Ouma, Guiyun Yan, James Kazura

**Affiliations:** 1 Department of Zoology, Maseno University, Kisumu, Kenya; 2 International Centre of Excellence for Malaria Research, Tom Mboya University College-University of California Irvine Joint Lab, Homa Bay, Kenya; 3 School of Natural and Environmental Science, Newcastle University, Newcastle Upon Tyne, UK; 4 Program in Public Health, University of California Irvine, Irvine, CA USA; 5 Centre for Global Health Research, Kenya Medical Research Institute, Kisumu, Kenya; 6 Department of Biomedical Sciences and Technology, Maseno University, Kisumu, Kenya; 7 Department of Pathology, School of Medicine, Case Western Reserve University, Cleveland, OH USA

**Keywords:** Active case detection, Malaria incidence, Ultrasensitive RDT, Health seeking behavior, Self-medication, Traditional medication, Kenya

## Abstract

**Background:**

Malaria remains a public health problem in Kenya despite sustained interventions deployed by the government. One of the major impediments to effective malaria control is a lack of accurate diagnosis and effective treatment. This study was conducted to assess clinical malaria incidence and treatment seeking profiles of febrile cases in western Kenya.

**Methods:**

Active case detection of malaria was carried out in three eco-epidemiologically distinct zones topologically characterized as lakeshore, hillside, and highland plateau in Kisumu County, western Kenya, from March 2020 to March 2021. Community Health Volunteers (CHVs) conducted biweekly visits to residents in their households to interview and examine for febrile illness. A febrile case was defined as an individual having fever (axillary temperature ≥ 37.5 °C) during examination or complaints of fever and other nonspecific malaria related symptoms 1–2 days before examination. Prior to the biweekly malaria testing by the CHVs, the participants' treatment seeking methods were based on their behaviors in response to febrile illness. In suspected malaria cases, finger-prick blood samples were taken and tested for malaria parasites with ultra-sensitive Alere^®^ malaria rapid diagnostic tests (RDT) and subjected to real-time polymerase chain reaction (RT-PCR) for quality control examination.

**Results:**

Of the total 5838 residents interviewed, 2205 residents had high temperature or reported febrile illness in the previous two days before the visit. Clinical malaria incidence (cases/1000people/month) was highest in the lakeshore zone (24.3), followed by the hillside (18.7) and the highland plateau zone (10.3). Clinical malaria incidence showed significant difference across gender (χ^2^ = 7.57; df = 2, *p* = 0.0227) and age group (χ^2^ = 58.34; df = 4, *p* < 0.0001). Treatment seeking patterns of malaria febrile cases showed significant difference with doing nothing (48.7%) and purchasing antimalarials from drug shops (38.1%) being the most common health-seeking pattern among the 2205 febrile residents (χ^2^ = 21.875; df = 4, *p* < 0.0001). Caregivers of 802 school-aged children aged 5–14 years with fever primarily sought treatment from drug shops (28.9%) and public hospitals (14.0%), with significant lower proportions of children receiving treatment from traditional medication (2.9%) and private hospital (4.4%) (*p* < 0.0001). There was no significant difference in care givers' treatment seeking patterns for feverish children under the age of five (*p* = 0.086). Residents with clinical malaria cases in the lakeshore and hillside zones sought treatment primarily from public hospitals (61.9%, 60/97) traditional medication (51.1%, 23/45) respectively (*p* < 0.0001). However, there was no significant difference in the treatment seeking patterns of highland plateau residents with clinical malaria (*p* = 0.431).The main factors associated with the decision to seek treatment were the travel distance to the health facility, the severity of the disease, confidence in the treatment, and affordability.

**Conclusion:**

Clinical malaria incidence remains highest in the Lakeshore (24.3cases/1000 people/month) despite high LLINs coverage (90%). The travel distance to the health facility, severity of disease and affordability were mainly associated with 80% of residents either self-medicating or doing nothing to alleviate their illness. The findings of this study suggest that the Ministry of Health should strengthen community case management of malaria by providing supportive supervision of community health volunteers to advocate for community awareness, early diagnosis, and treatment of malaria.

**Supplementary Information:**

The online version contains supplementary material available at 10.1186/s12879-022-07757-w.

## Background

Malaria remains a major public health problem in Kenya, despite increased efforts by the Ministry of Health to scale up intervention strategies [1–3]. Approximately 70% of the country's 47 million inhabitants are at risk of the disease, with the western Kenya region having the highest burden of infection [4]. The disease accounts for about 30% out-patients attendance in both public and private health care facilities [1, 2, 5, 6]. The most common symptom of clinical malaria is fever which drives people to seek treatment when it becomes severe [7]. Access to health care, housing type, proximity of human settlements to vector breeding sites [8], socioeconomic status, and bednet use [9, 10] may all influence clinical malaria risk in the community. Children and pregnant women are the most vulnerable groups to infection [11, 12]. School-aged children serve as a reservoir for malaria parasites, and the prevalence of infection among this age group is high [13]. Variation in the ecological landscape may result in differential risk exposures to malaria contributing to variation in febrile incidences in the community [14] driving residents to seek alternative treatment routes. Understanding the health-seeking behavior of clinical malaria cases across different topographical zones may aid in addressing the year-round infection in the community.

The Kenya National Guidelines for the Diagnosis, Treatment, and Prevention of Malaria call for seeking medical attention within 24 h of becoming ill [15]. Adherence to the guidelines, on the other hand, is becoming a problem as people suffering from malaria-related symptoms either self-medicate with over-the-counter medications or do nothing and only seek treatment from health facilities when their symptoms become severe. The COVID-19 pandemic has had a negative impact on fever care, with outpatient clinic attendance significantly lower since the virus was first detected in Kenya [16]. Prior to COVID-19, the majority of caregivers of children with fever sought treatment from public health facilities [15]. While the majority of parents seek medical attention for their feverish children, many do not do so right away. Some parents wait until their child's symptoms become severe before seeking medical help. Doing nothing about febrile symptoms, resulting in a delay in seeking treatment until the illness worsens, may lead to complicated malaria [17]. Although treatment in public health facilities is free, there is vast majority of underreported malaria cases at the community [18]. The availability and affordability of the local herbs is easier as they can be obtained from the fields or traditional healers as these traditional healers have good knowledge of symptoms of malaria [19–21]. The reasons for informing the decision to use the various treatment seeking rotes are unknown.

Self-medication for fever relief is a common practice, particularly in the early stages of illness when symptoms are mild [19]. Self-prescription and the use of antimalarial drugs without a confirmed diagnosis may result in antimalarial misuse, which may contributes to selection pressure [22]. In the absence of a confirmed diagnosis, alternative methods of treating malaria symptoms may result in disease complications. Monitoring the impact of topography on treatment seeking profiles in rural communities, as well as adherence to MOH-recommended prompt diagnosis and treatment guidelines, is critical for effective malaria control. As a result, more efforts must be made to encourage prompt fever treatment, as well as the adoption of more sensitive and accurate diagnostic tools to aid in community case management of malaria. The current study aims at assessing clinical malaria incidences and treatment seeking patterns across topography in a rural community in Kisumu County, western Kenya.

## Methods

### Study site

**Fig. 1:**
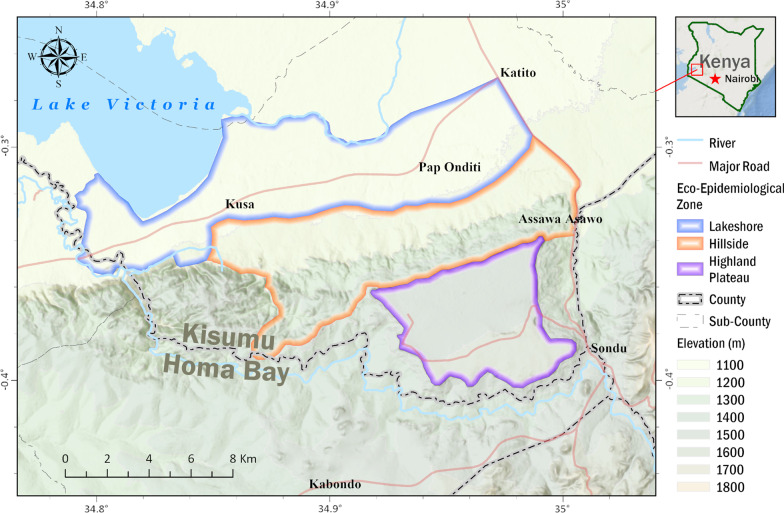
Map of Nyakach Sub-County in Kisumu County showing the study eco-epidemiological zones: Lakeshore zone (highlighted in blue), hillside zone (highlighted in brown), and highland plateau zone (purple highlighted)

This study was carried out in Nyakach Sub-County of Kisumu in Western Kenya. Based on malaria prevalence and topographical features, the study area was divided into three eco-epidemiological zones based on a previous study [18]: Lakeshore, Hillside, and Highland plateau (Fig. 1). Landscapes in the three zones are very different from each other based on altitude and topography [18]. The altitude of the three eco-epidemiological zones varies, with the lakeshore zone located on the lakeside of the Lake Victoria region at an altitude ranging from 1100 to 1200 m above sea level and prone to flooding during the rainy season. The Highland plateau zone has an altitude range of 1500–1700 m and has more stable larval habitats, whereas the Hillside zone has an altitude range of 1300–1450 m and is located between the Lakeshore and Highland plateau zones. Permanent aquatic habitats are uncommon and larval habitats unstable in this zone. The study area is approximately 327 square kilometers in size, with a population of 168,140 people living in 35,553 households at a population density of 460 people per square km [23]. This region's economic activities are primarily fishing, subsistence farming, rock mining, and small-scale trading.

### Study design and data collection

A longitudinal study was conducted in three eco-epidemiologically distinct zones: Lakeshore, Hillside, and Highland plateau zones, between March 2020 and March 2021.Community health volunteers (CHVs) were trained on how to record febrile cases in each household, as well as how to take blood sample for ultra-sensitive malaria RDT and RT-PCR analysis. The survey was conducted during the Covid-19 era, and all infection prevention and control protocols were followed in accordance with Ministry of Health guidelines [24]. A febrile suspected malaria case was defined as an individual with fever (axillary temperature ≥ 37.5 °C) at the time of examination or complaints of fever and other nonspecific symptoms within 48 h prior to examination, according to the WHO definition [25]. The survey sought to ascertain the community's health, demographic, and socioeconomic characteristics. The study questionnaire collected self-reported information on age, gender, and active fever, treatment seeking method prior to the CHVs testing, primary occupation, travel history, and ITN use. Participants were divided into three age groups (< 5 years old, 5–14 years old, and ≥ 15 years old). Active fever was defined as an individual axillary temperature ≥ 37.5 °C at the time of examination. The participants' treatment seeking methods were based on their behaviors in response to the illness prior to the biweekly malaria testing by the CHVs. There are five types of treatment seeking methods (public hospital, private hospital, drug shops, traditional medication, and do nothing) Occupation was divided into four categories (farmer, small scale business, office worker, unemployed, student, non-school child, and others. Travel history was defined as having traveled outside the study zones within the previous two weeks. ITN use was defined as sleeping under an ITN the night before the survey. The study sought to identify the socioeconomic and demographic factors associated with the decision to seek treatment. Furthermore, the study questionnaire gathered self-reported data on wall material type, health insurance, and malaria information and awareness, marital status, and distance to the health facility, severity of the disease, confidence in treatment choice, affordability, and medication availability. The wall material type was used to assess house structure categorized into the following groups: Brick/Block, Mud & Wood, and Mud & Cement. The health insurance was classified as the mode of payment at the health facility, as either the hospital payment was by health insurance or cash. Malaria information was defined as the medium through which participants learned about malaria prevention, symptoms, and control. Malaria information was divided into three categories: no information received, information received from the media, and information received from the CHV. Participants who knew the symptoms and severity of malaria were classified as "aware," while those who didn't know were classified as "not aware." The marital status was divided into four categories: under age, single, married, and widowed/divorced. The travel distance to the health facility, the severity of the disease, confidence in the choice of treatment, affordability, and medication availability were all evaluated to see if they played a role in treatment selection. These information was reviewed daily by team supervisors for quality assurance. A total of 2205 finger-prick blood samples were taken from febrile cases for parasite examination with ultra-sensitive Alere^®^ malaria RDT (Reference number: 05FK140, Republic of Korea) and RT-PCR on dry blood spots [26]. The samples were then transported to the International Centre of Excellence for Malaria Research (ICEMR) at Tom Mboya University College in Homa Bay, Kenya, for further analysis. The CHVs administered AL to all RDT positive febrile residents. Residents who tested negative were referred to the nearest health facility for follow-up care.

### DNA extraction and screening for *Plasmodium falciparum* infection

975 of the 2205 dried blood spots were randomly selected for DNA extraction to determine the sensitivity and specificity of the ultrasensitive malaria RDT. Chelex resin (Chelex-100) saponin method was used with slight modifications [26]. *Plasmodium* species-specific primers and probes targeting 18S ribosomal RNA were used [27]. PCR reaction volume was constituted as follows; 6 µL of PerfeCTa® qPCR ToughMix™, Low ROX™ Master mix (2X), 0.4 µL each of the forward and reverse species-specific primers (10 µM), 0.5 µL of the species-specific probe, 0.1 µL of double-distilled water and 2 µL of parasite DNA. Thermocycler conditions were set as follows, 50 °C for 2 min, (95 °C for 2 min, 95 °C for 3 s and 58 °C for 30 s) for 45 cycles (QuantStudio™ 3 Real-Time PCR System).

### Data analysis

Data were analyzed using SPSS Version 21 software. The demographic profiles of the study participants were described using descriptive statistics. The Chi-square test, odds ratio, incidence ratio, and risk ratio were used to identify the factors associated with clinical malaria incidences and the treatment seeking patterns. Multiple regression was used to predict malaria febrile incidence across topography. Artificial neural network model was used to identify the variables importance associated with the decision to seek treatment. Frequency tables were used to describe categorical variables (counts and percentages). For all analyses, *p* ≤ 0.05 was considered statistically significant.

## Results

### Demographic information of the study participants

A total of 1,599 households were surveyed, with 5,838 residents participating in the study. The three zones' residents’ age structure and gender were all similar. Farming was the most important source of income (21.7%). Individuals aged > 15 years made up approximately 56.6% of the study population and literacy rates were high, with 54.7% completing primary school and 26.2% completing secondary school education (Table 1).

**Table 1:** Descriptive statistics of the study participant’s demographic information

Parameter	Details	Enrollment	Eco-epidemiological zone (n, %)			P-value
			Lakeshore	Hillside	Plateau	
Total household surveyed		1599 (100.0)	460 (28.8)	501(31.3)	638 (39.9)	
Total enrolment		5838 (100.0)	1652 (28.3)	1605 (27.5)	2581 (44.2)	
Sex	Male	2728 (46.7)	749 (27.5)	774 (28.4)	1205 (44.1)	0.2554
	Female	3110 (53.3)	903 (29.1)	831(26.7)	1376 (44.2)	
Age	< 5	747 (12.8)	191(25.6)	209 (27.9)	347 (46.5)	0.1242
	5–14	1784 (30.6)	520 (29.1)	490 (27.5)	774 (43.4)	
	≥ 15	3307 (56.6)	941 (28.5)	906 (27.4)	1460 (44.1)	
Education	Never attended school	117 (2.0)	40 (34.2)	32 (27.4)	45 (38.4)	< 0.0001
	Pre-school age	639 (10.9)	130 (20.3)	189 (29.6)	320 (50.1)	
	Primary	3157 (54.7)	929 (29.4)	930 (57.9)	1298 (50.3)	
	Secondary	1531 (26.2)	426 (27.8)	373 (24.4)	732 (47.8)	
	College and above	394 (6.7)	127 (32.2)	81 (20.6)	186 (47.2)	
Occupation	Farmer	1029 (21.7)	222 (21.6)	316 (30.7)	491(47.7)	< 0.0001
	Small scale business	523 (11.0)	175 (33.5)	142 (27.2)	206 (39.3)	
	Office worker	138 (2.9)	62 (44.9)	36 (26.1)	40 (29.0)	
	Unemployed	489 (10.3)	132 (27.0)	169 (34.6)	188 (38.4)	
	Student	2879 (37.5)	897 (31.1)	762 (26.5)	1220 (42.4)	
	Non-school child	573 (12.1)	103 (18.0)	144 (25.1)	326 (56.9)	
	Others	207 (4.4)	61 (29.5)	36 (17.4)	110 (53.1)	

### Clinical malaria incidence across topography by age and gender

In the study zone, 2205 residents reported febrile illness out of a total of 5838 residents. The Lakeshore zone had the highest clinical malaria incidence, with 24.3 cases/1000 people/month, followed by the hillside zone (18.7 cases/1000 people/month) and the highland plateau zone (10.3 cases/1000 people/ month).

A further Chi square test revealed a statistically significant difference in clinical malaria incidence by gender across topographic zones (χ^2^ = 7.57; df = 2, *p* = 0.0227). Males had a higher incidence of 26.7 in the lakeshore zone than females, who had an incidence of 22.3. In the hillside and the highland plateau, the females had the higher incidence of infection at 21.7 and 12.7, respectively (Table 2).

**Table 2:** Malaria incidence/1000 people per month and incidence ratio across topography by age and gender

Parameters	Details	Lakeshore		Hillside		Highland plateau	
		Incidence	Incidence ratio (95% CI)	incidence	Incidence ratio (95% CI)	Incidence	Incidence ratio (95% CI)
Overall		24.3	Ref.^a^	18.7	0.77 (0.42–1.12)	10.3	0.42 (0.17–0.58)
Gender	Female	22.3	Ref.^b^	21.7	Ref.^b^	12.7	Ref.^b^
	Male	26.7	1.20 (0.74—1.65)	15.3	0.71 (0.35–1.06)	8.6	0.68 (0.22–1.13)
Age	< 5	34	Ref.^c^	17.2	Ref.^c^	13.4	Ref.^c^
	5–14	38.8	1.14 (0.78–1.50)	28.1	1.63 (1.03–2.24)	13.9	1.04 (0.49–1.58)
	≥ 15	11.7	0.34 (0.15–0.49)	12.9	0.75 (0.34–1.16)	7.9	0.59 (0.18–0.99)
Female	< 5	29.6	Ref.^d^	26.2	Ref.^d^	12.2	Ref.^d^
	5–14	50.8	1.72 (1.24–2.18)	32.7	1.25(0.82–1.67)	16.4	1.34 (0.69–1.99)
	≥ 15	15.1	0.51 (0.25–0.77)	14.9	0.57 (0.28–0.86)	10.8	0.89 (0.36–1.40)
Male	< 5	37.9	Ref.^e^	8.8	Ref.^e^	15	Ref.^e^
	5–14	48.2	1.27 (0.91–1.63)	23.4	2.66 (1.53–3.62)	12	0.80 (0.35–1.25)
	≥ 15	6.7	0.18 (0.04–0.31)	11.2	1.27 (0.51–1.95)	3.6	0.24 (0.01–0.49)

^a^Overall comparison was between survey zones using Lakeshore as reference

^b^Gender comparison was between sexes using females as references

^c^Age comparison was between the age groups using children < 5 years old as references

^d^Females comparison was between age groups using children < 5 years old as references

^e^Males comparison was between age groups using children < 5 years old as references

The chi square test revealed a significant difference in the incidence of clinical malaria by age group across topographical zones (χ^2^ = 58.34; df = 4, *p* < 0.0001). In the Lakeshore zone, hillside and the highland plateau the school going children aged between 5 and 14 years old had the highest incidence of infection at 38.8, 28.1, and 13.9 cases/1000 people/month, respectively (Table 2).

Among the females in the lakeshore zone, the risk of clinical malaria incidences was 1.72 times higher among the 5–14 years old school going children (IR:1.72, 95% CI = 1.24–2.18) and 0.51 times lower among individuals ≥ 15 years old (IR:0.51, 95% CI = 0.25–0.77) compared to children under 5 years old (Table 2). Among the males, the incidence risk of infection was 0.18 times lower among individuals ≥ 15 years old (IR: 0.18, 95% CI = 0.04–0.31) compared to children under five years old in the lake zone. In the hillside zone, the school going children had the highest incidence risk of infection compared to the children under five years old (IR: 2.66, 95% CI = 1.53–3.62) (Table 2).

### Risk factors associated with clinical malaria incidences

Multivariate analysis found that residency in the lakeshore and hillside zone, being male, being between the ages < 5 years and 5–14 years, having a subjective fever, and an elevated body temperature at the time of the visit were all associated with an increased risk of clinical malaria incidences (*p* < 0.05) (Table 3). When compared to the highland plateau, the odds of clinical malaria cases were 2.01(95% CI = 1.63–2.49, *p* < 0.0001) and 1.47 times higher in the lakeshore and hillside zones, respectively. Females were less likely than males to suffer from clinical malaria (OR: 0.83, 95% CI = 0.70–0.99, *p* = 0.042). When compared to individuals over the age of 15, school-aged children aged 5–14 years and under 5 years were 2.00 times (95% CI = 1.66–2.43, *p* < 0.0001) and 1.98 (95% CI = 1.54–2.54, *p* < 0.0001) more likely to suffer from clinical malaria, respectively. Residents who did not have active fever at the time of testing by the CHVs were less likely to test positive for malaria than those who did (OR: 0.27 95% CI = 0.21–0.34, *p* < 0.0001). However, seasonality, recent travel history, and bed net use were not associated with the risk of clinical malaria incidences (*p* < 0.05) (Table 3).

**Table 3:** Predictive factors associated with clinical malaria incidences

Risk factors	Category	Coefficient	Odd ratio (95% CI)^1^	*p*-value
Topographical zones	Lakeshore	0.698	2.01 (1.63, 2.49)	< 0.0001
	Hillside	0.386	1.47 (1.18, 1.84)	< 0.0001
	Highland plateau		1	
Gender	Female	− 0.182	0.83 (0.70, 0.99)	0.042
	Male		1	
Age group	< 5 years	0.683	1.98 (1.54, 2.54)	< 0.0001
	5–14 years	0.695	2.00 (1.66, 2.43)	< 0.0001
	≥ 15 years		1	
Temperature	< 37.5 °C	− 1.423	0.27 (0.21, 0.34)	< 0.0001
	≥ 37.5 °C		1	
Seasonality	Wet	− 0.07	0.93 (0.79–1.11)	0.427
	Dry		1	
Travel history	No	− 0.103	1.10 (0.69, 1.74)	0.693
	Yes		1	
Bed net usage	No net	0.087	0.78 (0.57, 1.07)	0.121
	Use net		1	

^1^Multivariate binary logistic regression model used for risk factor analysis

### Symptoms presented by ultrasensitive malaria RDT positive and negative residents

**Fig. 2:**
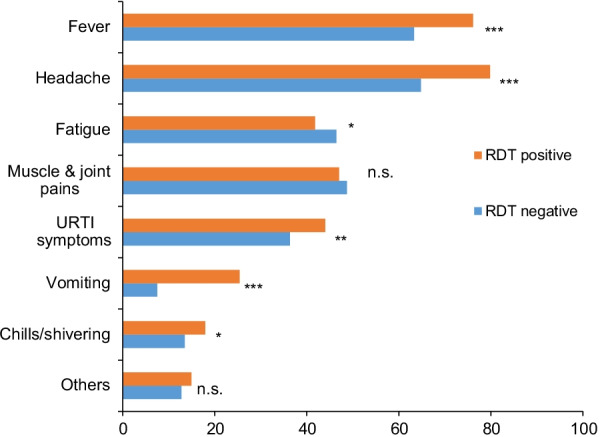
Percentage of symptoms presented by ultrasensitive malaria RDT positive and negative cases. Significance levels in difference between slide positive and negative groups for the same symptom: *P < 0.01, **P < 0.01, ***P < 0.001, and n.s. not significant at level of 0.05

The Chi square test revealed a significant difference in symptoms between residents who tested positive for malaria by ultrasensitive malaria RDT and those who tested negative (χ^2^ = 20.273, df = 7, *p* = 0.005). Fever, headache, chills, and vomiting were the most common symptoms among ultrasensitive malaria RDT positive residents, while fatigue, muscle and joint pain were common among ultrasensitive malaria RDT negative residents (Fig. 2).

**Fig. 3:**
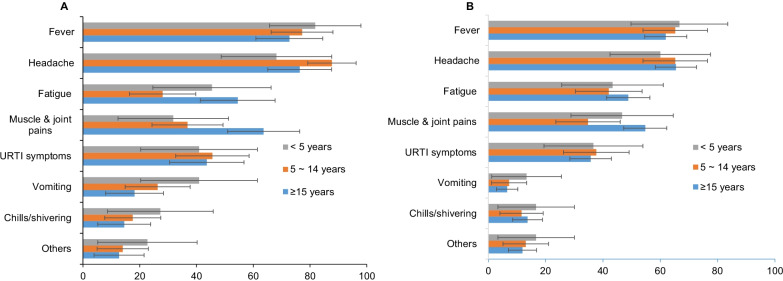
**A** Percentage of symptoms of ultrasensitive malaria RDT positive within the age groups. **B** Percentage of symptoms of ultrasensitive malaria RDT negative cases within the age groups

The Chi square test revealed no significant differences in the symptoms reported by ultrasensitive malaria RDT positive residents across age groups (χ^2^ = 16.537, df = 14, *p* = 0.282). Furthermore, the test revealed no significant difference in symptoms among those who tested negative for ultrasensitive malaria RDT across age groups (χ^2^ = 6.577, df = 14, *p* = 0.950) (Fig. 3).

### Treatment seeking patterns of clinical malaria cases

The identified treatment seeking patterns of the clinical malaria cases were doing nothing, buying medicine from drug stores/chemists, and seeking treatment in public, private, and traditional medication which was mainly herbal remedies. There was significant difference in the treatment seeking profiles of the clinical malaria cases (χ^2^ = 21.875; df = 4, *p* < 0.0001). The most common health seeking behavior among the total 2205 febrile cases assessed was doing nothing (48.7%), buying medicine from drug shops/chemists (38.1%), and seeking treatment in public (12.5%), private hospitals (4.1%), and traditional medication (3.5%) (Table 4). Treatment seeking patterns for the clinical malaria cases differed significantly by the lakeshore (χ^2^ = 22.471, df = 4, *p* < 0.0001), hillside zones (χ^2^ = 27.813, df = 4, *p* < 0.0001), female sex (χ^2^ = 19.447, df = 4, *p* = 0.001), school going children (χ^2^ = 21.717, df = 4, *p* < 0.0001), residents with active fever (temperature ≥ 37.5 °C) at the time of visit (χ^2^ = 11.943, df = 4, *p* = 0.018), bednet users(χ^2^ = 16.355, df = 4, *p* = 0.003), and bednet non users (χ^2^ = 15.945, df = 4, *p* = 0.003) (Table 4). 28.9% (232/802) of caregivers of school-aged children aged 5 to 14 years old with fever sought treatment from drug shops, while 14.0% (112/802) sought treatment from public health facilities, with much lower proportions of children receiving fever treatment from traditional medication (2.9%, 23/802) and private health facility (4.4%, 35/802) (*p* < 0.0001). However, There was no significant difference in care givers' treatment seeking patterns for feverish children under the age of five (*p* = 0.086). Although the majority of children receive fever treatment at government facilities, the proportion of children seeking treatment varies by topography (*p* < 0.0001).

**Table 4:** Descriptive statistics of clinical malaria cases treatment seeking method

Items	Category	Enrolment		Treatment seeking method										χ^2^-value	p-value
				Public hospital		Private hospital		Drug shop		Traditional medication		Do nothing			
Subjects		Fever† N (%)	Pos.‡ n (%)	Fever† N (%)	Pos.‡ (%)	Fever† n (%)	Pos.‡	Fever† n (%)	Pos.‡	Fever† n (%)	Pos.‡	Fever† n (%)	Pos.‡		
Overall	N	2205	850 (38.5)	275 (12.5)	115 (41.8)	91 (4.1)	15 (16.5)	688 (31.2)	248 (38.1)	77 (3.5)	28 (45.5)	1074 (48.7)	387 (39.4)	21.875	< 0.0001
Zones	Lakeshore	811 (36.8)	374 (46.1)	97 (12.0)	60 (61.9)	44 (5.4)	10 (22.7)	146 (18.0)	75 (51.4)	16 (2.0)	8 (50.0)	508 (62.6)	221 (43.5)	22.471	< 0.0001
	Hillside	691 (31.3)	266 (38.5)	88 (12.7)	21 (23.9)	36 (5.2)	3 (8.3)	263 (38.1)	107 (40.7)	45 (6.5)	23 (51.1)	259 (37.5)	112 (43.2)	27.813	< 0.0001
	Plateau	703 (31.9)	210 (29.9)	90 (12.8)	34 (37.8)	11 (1.6)	2 (18.2)	279 (39.7)	80 (28.7)	16 (2.3)	4 (25.0)	307 (43.7)	90 (29.3)	3.821	0.431
Sex	Male	857 (38.9)	353 (41.2)	129 (15.1)	51 (39.5)	37 (4.3)	7 (18.9)	266 (31.0)	114 (42.9)	24 (2.8)	8 (33.3)	401 (46.8)	173 (43.1)	9.270	0.055
	Female	1348 (61.1)	497 (36.9)	146 (10.8)	64 (43.8)	54 (4.0)	8 (14.8)	422 (31.3)	148 (35.1)	53 (3.9)	27 (50.9)	673 (49.9)	250 (37.1)	19.447	0.001
Age	< 5	345 (15.7)	159 (46.1)	48 (13.9)	23 (47.9)	13 (3.8)	4 (30.8)	106 (30.7)	57 (53.8)	15 (4.3)	3 (20.9)	163 (47.2)	72 (44.2)	8.612	0.086
	5 ~ 14	802 (36.3)	372 (46.4)	112 (14.0)	58 (51.8)	35 (4.4)	4 (11.4)	232 (28.9)	102 (44.0)	23 (2.9)	14 (60.9)	400 (49.9)	194 (48.5)	21.717	< 0.0001
	≥ 15	1058 (48.0)	319 (30.1)	115 (10.9)	34 (29.6)	43 (4.1)	7 (16.3)	350 (33.1)	103 (29.4)	39 (3.7)	18 (46.2)	511 (48.3)	157 (30.7)	8.856	0.065
Temperature	< 37.5 °C	1833 (83.1)	601(32.8)	214 (11.7)	65 (30.4)	78 (4.3)	10 (12.8)	591 (32.2)	201 (34.0)	67 (3.7)	29 (87.0)	883 (48.2)	296 (33.5)	18.643	0.001
	≥ 37.5 °C	372 (16.9)	249 (66.9)	61 (16.4)	50 (82.0)	13 (3.5)	5 (38.5)	97 (26.1)	61 (62.9)	10 (2.7)	6 (13.0)	191 (51.3)	127 (66.5)	11.943	0.018
Bednet Usage	Yes	1987 (90.1)	770 (38.8)	253 (12.7)	111 (43.9)	78 (3.9)	15 (19.2)	610 (30.0)	232 (38.0)	75 (3.8)	33 (44.0)	971 (48.9)	379 (39.0)	16.355	0.003
	No	218 (9.9)	80 (36.7)	22 (10.1)	4 (18.2)	13 (6.0)	0	78 (35.8)	30 (38.5)	2 (0.9)	2 (100)	103 (47.2)	44 (42.7)	15.945	0.003

Degree of freedom (d.f) = 4

†n (%) ‡percentage

Residents with clinical malaria cases in the lakeshore zones sought treatment primarily from public hospitals (61.9%, 60/97) and purchased medication from drug shops (51.4%, 75/146) (*p* < 0.0001). Residents in the hillside zone with clinical malaria cases sought treatment primarily through traditional medication (51.1%, 23/45) (*p* < 0.0001). There was, however, no significant difference in treatment seeking patterns between Highland plateau residents with clinical malaria cases (*p* = 0.431) (Table 4).

Females with clinical malaria sought treatment primarily from traditional medications (50.9%, 27/53) and at public hospitals (43.8%, 64/146). Children, on the other hand, are unable to make treatment decisions on their own, and their treatment seeking pattern is determined by their parents/guardians (Table 4).

Majority of the clinical malaria cases were from school going children under traditional medication (60.9%, 14/23) and at the public hospital (51.8%, 58/112). Of the residents with active fever at the time of visits, clinical malaria cases were mostly from those who sought prior treatment from: public hospital (82.0%, 50/61) and those who did nothing (66.5%, 127/191) (Table 4).

### Factors associated with decision to seek treatment

Artificial neural network model was used to identify the main factors associated with the decision to seek treatment. Independent variable importance analysis showed that distance (Importance = 0.184, normalized importance = 100%), severity of disease (Importance = 0.163, normalized importance = 88.7%), confidence in the treatment (Importance = 0.108, normalized importance = 58.5%), affordability (Importance = 0.100, normalized importance = 54.4%), availability of medication (Importance = 0.090, normalized importance = 49.1%), marital status (Importance = 0.072, normalized importance = 39.2%), health insurance (Importance = 0.057, normalized importance = 30.8%), malaria awareness (Importance = 0.054, normalized importance = 29.50%), socio-economic status (wall type: Importance = 0.054, normalized importance = 29.4% and floor type: Importance = 0.038, normalized importance = 20.70%), Knowledge of malaria (Importance = 0.034, normalized importance = 18.8%), net usage (Importance = 0.028, normalized importance = 15.0%) and gender (Importance = 0.018, normalized importance = 10.0%) were the main factors associated with decision to seek treatment. (Training: cross entropy error = 167.712, incorrect prediction = 25.1%; Testing: cross entropy error = 110.675; incorrect prediction = 35.4%) (Additional file 1: Table S1).

A subsequent analysis revealed a significant relationship between treatment seeking pattern and distance to the health facility (χ^2^ = 98.816, df = 4, *p* < 0.0001). Residents who reported distance to the health facility as a factor in their decision to seek treatment did nothing (34.4%, 65/188). Residents who reported that distance was not a factor in their decision to seek treatment, on the other hand, primarily sought treatment from private (31.6%, 67/212) and public hospitals (27.4%, 58/212) (Additional file 1: Table S2).

The severity of the diseases was significantly related to the treatment seeking preference (χ^2^ = 121.246, df = 4, *p* < 0.0001). Residents who said the severity of the malaria disease influenced their decision to seek treatment did so primarily through traditional medicine (26.2%, 67/256) and private hospitals (25.8%, 66/256). Residents who reported that the severity of the diseases was not a factor in their decision to seek treatment, on the other hand, largely did nothing (48.6%, 70/144) (Additional file 1: Table S2).

Residents' confidence in their treatment options was significantly related to their decision to seek treatment (χ^2^ = 33.442, df = 4, *p* < 0.0001). The majority of residents were confident in seeking treatment in a public hospital (25.1%, 55/219), traditional medication (24.7, 54/219), and private hospital (20.1%, 44/219). Residents who did nothing (31.5%, 57/181) and bought drugs from drug shops (20.4%, 37/181), on the other hand, reported having little confidence in their treatment option (Additional file 1: Table S2).

The cost of the medication had a significant impact on the decision to seek treatment. (χ^2^ = 80.640, df = 4, *p* < 0.0001). The majority of residents could easily afford treatment from traditional medications (27.2%, 68/250) and public hospitals (25.6%, 64/250). On the other hand, most residents could not afford medication from private hospitals (38.7%, 58/150) (Additional file 1: Table S2).

Furthermore, the availability of medication was strongly related to the decision to seek treatment (χ^2^ = 93.594, df = 4, *p* < 0.0001). The majority of residents stated that medication was easily accessible from traditional medication (29.4%, 67/228), drug shops (27.6%, 63/228), and private hospitals (20.6%, 47/228). Those who did nothing (38.4%, 66/172) and those who sought medication from public hospitals (25.0%, 43/172) both reported that medication was not readily available (Additional file 1: Table S2).

## Discussion

The current study looked at clinical malaria incidences, treatment seeking profiles of febrile cases and factors associated with the decision to seek treatment in western Kenya. Clinical malaria incidence (cases/1000people/month) was highest in the lakeshore zone (24.3), followed by the hillside (18.7) and the highland plateau zone (10.3). The most common health-seeking behaviors among the 2205 febrile residents were doing nothing (48.7%) and purchasing antimalarial from local drug shops (38.1%). Malaria was present in the residents who did nothing, sought traditional medication, and purchased antimalarial from drug shops, and from public hospitals at the time of the visit. The decision to seek treatment was heavily influenced by the distance to the health facility, the severity of the disease, confidence in the treatment, affordability, and socioeconomic status. Furthermore, the current study observed that ultrasensitive malaria RDT diagnosis is highly specific (98.7%) and has good sensitivity (65.5%).

The high clinical malaria incidences and the positivity rates in the Lakeshore zone may be attributed to the area's flat plains and frequent flooding during the rainy seasons, resulting in water stagnation and the presence of permanent mosquito breeding habitats, as well as households' proximity to open water sources, which are stable larval habitats and potential mosquito breeding grounds. The findings corroborate previous research from western Kenya that found a high prevalence of malaria along the lake basin [28–33]. The primary economic activities in the current study region are subsistence farming and small-scale businesses such as fishing and rock mining. Residents' economic activities, such as night fishing and dusk small-scale businesses, may cause them to remain outside without protective measures, exposing themselves to mosquito bites. However, the current study did not investigate the relationship between economic activity and malaria burden.

In the current study, male in the lakeshore zones were more likely to contract malaria than females. This could be attributed to socioeconomic differences, with the majority of adult males engaging in nighttime outdoor activities that expose them to mosquito bites if no protective measures are taken [34, 35]. Females, on the other hand, were more likely to contract malaria in the hillside and highland plateau zones, most likely as a result of dusk activities such as selling vegetables and outdoor cooking at night, which exposes them to mosquito bites [36]. Females have pre-natal clinic appointments during pregnancy and frequently take their children to seek treatment, which may explain their high hospital seeking behavior and, as a result, their lower clinical malaria incidences when compared to males [37, 38]. Clinical malaria incidences were high among school-aged children aged 5–14 years in all study zones, according to the findings. Lower bednet usage among school-aged children exposes them to high mosquito bites at night, which may explain why clinical malaria incidences are higher in this age group [34, 39, 40]. The low infection rate among children under the age of five compared to school-age children could be attributed to the children being cared for by their parents and sleeping under mosquito nets at night [12, 41–43]. Children who sleep under insecticide-treated mosquito nets were less likely to contract malaria than those who did not sleep under bednets [41]. A similar study in Mozambique discovered that self-reported symptomatic malaria is extremely common among children, and that factors facilitating access to health care are associated with symptomatic malaria diagnosis [7]. Individuals in malaria-endemic areas develop adaptive immunity to the *P. falciparum* parasite, resulting in a decreasing rate of infection with age [44]. Similarly, a study in Burkina Faso linked increased fever cases among children to malaria infection (27). The current study, on the other hand, found that health seeking profiles did not differ by age group. Children, unlike adults, are unable to make treatment decisions on their own because their parents or guardians determine the treatment pattern [37, 38].

Individuals suspected of having malaria often start by doing nothing, then self-medicate with drugs from drug stores or traditional medications, and when the condition worsens, they seek treatment at health facilities. Doing nothing was most commonly reported among febrile residents, but when their febrile condition worsened, these residents were more likely to seek other alternative treatment options. In the current study, more than 80% of residents either self-medicate or do nothing when they have febrile illness, with only less than 20% seeking treatment in a health facility. In the current study, less than 20% of residents were reported to seek malaria treatment in health facilities, with an estimated 80% of febrile cases being underreported, with a proportion of whom could be malaria cases not being recorded in health facilities. A large proportion of the community does not seek treatment at health care facilities for a variety of reasons, including a lack of antimalarial in health facilities, the affordability of malaria diagnosis and distance to health facilities, confidence in the treatment, and socioeconomic status. As a result, approximately 31% of febrile cases self-diagnose and self-treat with drugs obtained from local drug shops located in nearly every shopping center. A Nigerian study found that approximately 88% of residents prefer to manage malaria at home, with only about 12% visiting health facilities [17]. The use of antimalarial drugs in the absence of a confirmed test is a major source of concern. Despite seeking treatment from drug stores and traditional medication, inappropriate treatment may have contributed to the observed higher clinical malaria cases in the current study.

Traditional medicine is commonly used to treat fever in African communities, especially during the early stages of illness or when the symptoms are mild [17, 21, 45]. According to the current study, the hillside zone, which is mostly hilly and has a lot of herbs and shrub plantation, explains why the majority of the residents are more likely to seek traditional medicine. Local herbs are more accessible and affordable because they can be obtained from the fields or traditional healers. According to studies in the Democratic Republic of the Congo, Guinea, and Kenya, traditional healers have a good understanding of malaria symptoms and causes, resulting in consistent knowledge of antimalarial plants [19–21]. It has been reported that herbal medications are involved in parasite clearance [17, 21, 45]. Furthermore, healthcare facilities in the hillside zone were scarce, which could explain the decision to seek traditional medication form of treatment. The current study, however, did correlate the availability of health facilities across topographies and treatment seeking profiles. The current study, however, did not follow up on the parasite clearance by traditional herbs. The study showed socio-economic status such as that the type of housing wall and the floor type, distance to medication access, and hospital payment method all influenced the decision to seek treatment. Residents from the lake zone, for example, were more likely to seek treatment in a public hospital and purchase antimalarial drugs from local drug stores. This was greatly influenced by the distance and ease of access. The severity of fever as a result of *P. falciparum* infection drives people to seek treatment [7] which is heavily influenced by accessibility, availability, and affordability of treatment services [22]. The current study residents reported taking analgesics to relieve pain before taking antimalarials, which may explain why there were fewer active fever cases in the study zone.

The rapid emergence and spread of the COVID-19 has resulted in massive global disruptions that are affecting people's lives and well-being. The devastation caused by the pandemic could be greatly exacerbated if the response jeopardizes the provision of life-saving malaria services [46]. COVID-19-related challenges have contributed to an increase in antimalarial and RDT stockout rates, resulting in a drop in test-and-treat policy adherence [16]. Reduced funding for vector interventions, combined with competing public health challenges such as the ongoing COVID-19 pandemic, may result in a rollback of malaria control gains, leading to increased morbidity and mortality from malaria [47–49]. Furthermore, fear and stigma were generated as a result of the COVID-19 situation. Fear of contracting COVID-19 in a health facility, for example, as well as the stigma of being tested for COVID-19 infection, influenced facility attendance. The number of people visiting health-care facilities decreased as a result of such concerns. The Kenya malaria indicator survey has also reported similar findings [50].

According to the current study, ultrasensitive malaria RDT diagnosis had a higher specificity (99%) and a good sensitivity (66%) in detecting malaria febrile cases. The study's findings confirm the high sensitivity of the ultrasensitive malaria RDT when compared to RT-PCR, as previously reported [51–53]. Malaria intervention strategies are dependent on whether malaria patients can easily access and afford appropriate diagnosis and treatment. To reduce the complication of malaria cases, the government should invest in supportive supervision of CHVs as well as the provision of more sensitive RDTs and antimalarial to strengthen community malaria case management.

## Conclusion

Malaria case treatment-seeking habit is critical in determining malaria infection at the community level. Despite high bednet coverage, the current study found that the community has a high rate of clinical malaria incidences and positivity rates with the lakeshore zones bearing the greatest burden. The number of febrile cases is high because only about 20% of residents seek diagnosis and treatment in health care facilities, while the other 80% self-medicate or do nothing. These health seeking behavior suggests that a portion of the community's reported 80% of febrile cases may be infected with malaria but not reported in the Kisumu's monthly DHIS-2 reporting system. More research should be done to determine the true number of malaria-infected people who aren't reported in the DHIS-2. This information will help the Ministry of Health strengthen its community case management strategy for malaria.

## Supplementary Information

**Additional file 1: Table S1**. Independent variable importance associated with decision to seek treatment. **Table S2**. Factors associated with decision to seek treatment.

## Data Availability

The dataset used in this study is available from the corresponding author upon request.

## Abbreviations

CI: Confidence Interval
CHV: Community Health Volunteer
DBS: Dried blood spots
DNA: Deoxyribonucleic acid
ICEMR: International Center of Excellence for Malaria Research
RDT: Rapid diagnosis test
RT-PCR: Real-time polymerase chain reaction
