# Supplementary material for: Clinical malaria incidence and health seeking pattern in geographically heterogeneous landscape of western Kenya
Source: BMC Infect Dis. 2022 Oct 3;22:768. doi: 10.1186/s12879-022-07757-w (PMC9528858; doi:10.1186/s12879-022-07757-w)
Supplement: Supplementary file 1 — Additional file 1: Table S1. Independent variable importance associated with decision to seek treatment. Table S2. Factors associated with decision to seek treatment. [file 12879_2022_7757_MOESM1_ESM.docx]

**S1 Table. Independent variable importance associated with decision to seek treatment**

| **Independent Variable Importance** | | | **Predicted** | | | | | | | | **Model summary** | |
| --- | --- | --- | --- | --- | --- | --- | --- | --- | --- | --- | --- | --- |
| **Details** | **Importance** | **Normalized Importance** | **Sample** | **Observed** | **Do nothing** | **Traditional medication** | **Drug shop** | **Public hospital** | **Private hospital** | **Percent correct** | **Cross**  **entropy error** | **Incorrect prediction (%)** |
| Distance to health facility | 0.184 | 100.00% | **Training** | Do nothing | 45 | 0 | 2 | 4 | 0 | 88.20% | 167.712 | 25.1 |
| Severity of disease | 0.163 | 88.70% |  | Traditional medication | 0 | 44 | 8 | 5 | 7 | 68.80% |  |  |
| Confidence in treatment choice | 0.108 | 58.50% |  | Drug shop | 4 | 16 | 33 | 0 | 0 | 62.30% |  |  |
| Affordability | 0.1 | 54.40% |  | Public hospital | 4 | 0 | 2 | 48 | 5 | 81.40% |  |  |
| Medication availability | 0.09 | 49.10% |  | Private hospital | 1 | 3 | 5 | 3 | 36 | 75.00% |  |  |
| Marital status | 0.072 | 39.20% |  | Overall percent | 19.60% | 22.90% | 18.20% | 21.80% | 17.50% | 74.90% |  |  |
| Health insurance | 0.057 | 30.80% | **Testing** | Do nothing | 21 | 1 | 3 | 4 | 0 | 72.40% | 110.675 | 35.4 |
| Malaria awareness | 0.054 | 29.50% |  | Traditional medication | 0 | 8 | 3 | 2 | 3 | 50.00% |  |  |
| Wall type | 0.054 | 29.40% |  | Drug shop | 2 | 7 | 18 | 0 | 0 | 66.70% |  |  |
| Floor type | 0.038 | 20.70% |  | Public hospital | 2 | 0 | 1 | 14 | 4 | 66.70% |  |  |
| Malaria knowledge | 0.034 | 18.80% |  | Private hospital | 0 | 0 | 9 | 2 | 21 | 65.60% |  |  |
| Net usage | 0.028 | 15.00% |  | Overall percent | 20.00% | 12.80% | 27.20% | 17.60% | 22.40% | 65.60% |  |  |
| Gender | 0.018 | 10.00% |  |  |  |  |  |  |  |  |  |  |

Dependent variable: Treatment seeking type

**S2 Table. Factors associated with decision to seek treatment**

| **Decisions** | **Details** | **Enrolment** | **Public hospital** | **Private hospital** | **Drug shop** | **Traditional methods** | **Do nothing** | χ^2^ | df | p-value |
| --- | --- | --- | --- | --- | --- | --- | --- | --- | --- | --- |
| **Distance to heath facility** | yes | 188 (47.0) | 22 (11.7) | 13 (6.9) | 56 (29.8) | 32 (17.0) | 65 (34.4) |  | 4 | <0.0001 |
|  | no | 212 (53.0) | 58 (27.4) | 67 (31.6) | 24 (11.3) | 48 (22.6) | 15 (7.1) | 98.816 |  |  |
| **Severity of disease** | Yes | 256 (64.0) | 59 (23.0) | 66 (25.8) | 54 (21.1) | 67 (26.2) | 10 (3.9) |  |  | <0.0001 |
|  | No | 144 (36.0) | 21 (14.6) | 14 (9.7) | 26 (18.1) | 13 (9.0) | 70 (48.6) | 121.246 | 4 |  |
| **Confidence in choice of treatment** | yes | 219 (54.8) | 55 (25.1) | 44 (20.1) | 43 (19.6) | 54 (24.7) | 23 (10.5) | 33.442 | 4 | <0.0001 |
|  | no | 181 (45.3) | 25 (13.8) | 36 (19.9) | 37 (20.4) | 26 (14.4) | 57 (31.5) |  |  |  |
| **Affordable** | yes | 250 (62.5) | 64 (25.6) | 22 (8.8) | 38 (15.2) | 68 (27.2) | 58 (23.2) | 80.640 | 4 | <0.0001 |
|  | no | 150 (37.5) | 16 (10.7) | 58 (38.7) | 42 (28.0) | 12 (8.0) | 22 (14.7) |  |  |  |
| **Availability of medication** | yes | 228 (57.0) | 37 (16.2) | 47 (20.6) | 63 (27.6) | 67 (29.4) | 14 (6.1) | 93.594 | 4 | <0.0001 |
|  | no | 172 (43.0) | 43 (25.0) | 33 (19.2) | 17 (9.9) | 13 (7.6) | 66 (38.4) |  |  |  |
